# The Progress in the Treatment of Hepatocellular Carcinoma With Portal Vein Tumor Thrombus

**DOI:** 10.3389/fonc.2021.635731

**Published:** 2021-09-23

**Authors:** Fangzhou Luo, Mengxia Li, Jun Ding, Shusen Zheng

**Affiliations:** ^1^ Division of Hepatobiliary and Pancreatic Surgery, Department of surgery, The First Affiliated Hospital, Zhejiang University School of Medicine, Hangzhou, China; ^2^ School of Medicine, Zhejiang University, Hangzhou, China; ^3^ NHC Key Laboratory of Combined Multi-Organ Transplantation, Hangzhou, China; ^4^ Key Laboratory of the Diagnosis and Treatment of Organ Transplantation, Research Unit of Collaborative Diagnosis and Treatment For Hepatobiliary and Pancreatic Cancer, Chinese Academy of Medical Sciences, Hangzhou, China; ^5^ Key Laboratory of Organ Transplantation, Research Center for Diagnosis and Treatment of Hepatobiliary Diseases, Hangzhou, China

**Keywords:** hepatocellular carcinoma, portal vein tumor thrombus, transarterial chemoembolization, radiotherapy, liver transplantation, targeted therapy

## Abstract

Hepatocellular carcinoma (HCC) is one of most prevalent cancer and is a serious healthcare issue worldwide. Portal vein tumor thrombus (PVTT) is a frequent complication and remains as the blockage in the treatment of HCC with high recurrence rate and poor prognosis. There is still no global consensus or standard guideline on the management of HCC with PVTT. In western countries, Sorafenib and Lenvatinib are recommended as the first-line treatment options for HCC patients with PVTT where this condition is now regarded as BCLC Stage C regardless of PVTT types. However, there is growing evidence that supports the close relationship of the extent of PVTT to the prognosis of HCC. Besides the targeted therapy, more aggressive treatment modalities have been proposed and practiced in the clinic which may improve the prognosis of HCC patients with PVTT and prolong the patients’ survival time, such as transarterial chemoembolization, radiotherapy, hepatic resection, liver transplantation, and various combination therapies. Herein, we aim to review and summarize the advances in the treatment of HCC with PVTT.

## Introduction

Liver cancer is the sixth most commonly diagnosed cancer and the fourth cause of cancer-related deaths worldwide (1). In the last decade, the incidence and mortality of liver cancer keep increasing rapidly (2–4). In 2008, an estimated number of 748,300 new liver cancer cases and 695,900 deaths occurred globally (2). According to global cancer statistics, nearly 841,000 new liver cancer cases and 782,000 deaths were estimated to occur in 2018 (4).

Hepatocellular carcinoma (HCC) is the major histological subtype, accounting for 75% – 85% of cases among the primary liver cancers, while intrahepatic cholangiocarcinoma and other rare types only account for 10% – 15% of cases (4). The symptoms of early HCC are often imperceptible, and about 70% – 80% of patients are already in the advanced stage at the time of diagnosis (5, 6). The overall outcome of HCC still remains unsatisfactory, especially when the HCC is accompanied by the invasion of intrahepatic vessels (the portal vein or hepatic vein branches). It is one of the most common complications of advanced HCC and has been proven to be closely related with the poor prognosis (7).

Portal vein tumor thrombus (PVTT) is the most frequent form of macrovascular invasion that occurs in 44.0% – 62.2% of HCC patients (8), while the incidence of hepatic vein tumor thrombus (HVTT) (1.4% – 4.9%) (9) or the inferior vena cava/intra-right atrial tumor thrombus (3% – 4%) is rare (10). Llovet et al. (11) analyzed the natural history of HCC patients associated with PVTT and reported that the median survival time (MST) was only 2.7 months without treatment. Giannelli et al. (12) retrospectively analyzed 150 HCC patients and found that the occurrence of PVTT was the most important and reliable negative prognostic factor (P<0.01). Recently, Mahringer-Kunz et al. (13) carried out a retrospective cohort study of 1317 HCC patients. The results showed that 484 patients presented with PVTT and it counted for 36.8% of the cases. The MST of patients with PVTT was 7.2 months, which was significantly shorter than the patients without PVTT (35.7 months, P < 0.001). The study found that the degree of PVTT is not a determined factor, because even the minor PVTT could lead to a very poor prognosis of HCC patients. Taken together, PVTT is an independent risk factor and associated with a dismal prognosis in HCC patients.

At present, there is still no global consensus or standard guidelines on the management of HCC with PVTT. According to the Barcelona Clinic for Liver Cancer (BCLC) staging system and treatment guidelines which are widely used in Europe and America, HCC patients with PVTT are regarded as BCLC Stage C which strongly indicates an advanced stage of the disease (7, 14–17). These guidelines recommend Sorafenib as the standard first-line treatment option but the effect is modest (18). In recent years, Lenvatinib was also approved and recommended as the first-line therapy for HCC (7). In order to improve the prognosis of HCC patients with PVTT, the more aggressive treatment modalities have been proposed in the Asia–Pacific region (6, 19, 20). Besides the small molecular targeted therapy, transarterial chemoembolization (TACE), radiotherapy (RT), hepatic resection, and liver transplantation (LT) have been practiced in the clinical and recognized gradually. Herein, we aim to review and summarize the advances in the diagnosis and treatment of HCC with PVTT.

## Diagnosis and Classification of PVTT

On the basis of the diagnosis of HCC, we need to distinguish PVTT from Portal vein thrombus (PVT) which usually occurred in cirrhosis patients and is important for the selection of treatment and the prognosis of HCC. Pathological analysis remains the gold standard to diagnose PVTT so far, but the clinical diagnosis mainly relies on computed tomography (CT) scan and magnetic resonance imaging (MRI) (21, 22). Kim et al. (23) retrospectively analyzed the gadoxetic acid–enhanced MR imaging of 366 HCC patients, and found that the characteristic imaging features of PVTT group were the enhancement, vessel expansion, continuity of the tumor, increased T2 signal intensity, and diffusion restriction. Agarwal et al. (24) presented a case report and put forward that ^18^F-FDG PET/CT scan has good diagnostic performance in differentiating the malignant from benign thrombus. This view was subsequently validated by Wu et al. (25). Recently, by evaluating the radiographic features and clinical characteristics, Sherman et al. (26) found that the alpha-fetoprotein (AFP) >1000 ng/dL, venous expansion, thrombus enhancement, neovascularity, and adjacent to HCC were the characteristics of PVTT. They further proposed a noninvasive diagnostic criterion named the A-VENA criteria. The presence of 3 or more of these criteria could accurately differentiate PVTT from PVT (26).

**Figure 1:**
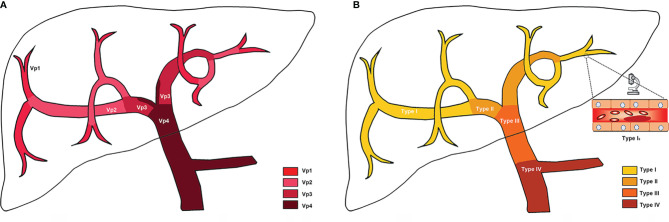
Schematic of PVTT classifications. **(A)** Japanese Vp classification; **(B)** Chinese Cheng’s classification. More details of classifications have been listed in **Table 1**.

The prognosis of HCC is not only related to the existence of PVTT, but also closely related to the extent of PVTT (27). Various classification systems for PVTT have been developed in different centers (28–32). Currently, there are two PVTT classification systems which are widely used in clinical practice (**Table 1**, **Figure 1**). The Japanese Vp classification (28, 33) is the first PVTT classification system which comprises five grades based on the extent of PVTT: 1) Vp0 for no PVTT; 2) Vp1 for tumor thrombus involving segmental PV; 3) Vp2 for tumor thrombus involving the second-order branches of PV; 4) Vp3 for tumor thrombus involving the first-order branches of PV; and 5) Vp4 for tumor thrombus involving the main trunk and/or contralateral branch of PV. In the Asia-Pacific, the more applicable classification system is the Chinese Cheng’s classification (29, 30). It classifies PVTT macroscopically into four types based on the medical imaging results: 1) Type I, the tumor thrombus invades segmental PV or above. If the postoperative pathological result shows that the tumor thrombus is confined to microvascular, it is classified as Type I_0_; 2) Type II, the tumor thrombus invades the right or/and left PV; 3) Type III, the tumor thrombus invades the main PV; and 4) Type IV, the tumor thrombus invades the superior mesenteric vein. Recently, Cao et al. (34) proposed a decision tree algorithm-based classification system by comprehensively considering both the extent of PVTT and HVTT, and generated 13 vascular invasion sub-classes. The classification system enables to personalize the management of HCC patients with vascular invasion, but its performance needs further assessment in more clinical studies.

**Table 1:** Classifications of PVTT.

Extent of tumor thrombus	Japanese Vp classification	Chinese Cheng’s classification
no PVTT	Vp0	NA
microvascular	NA	Type I_0_
segmental PV or above	Vp1	Type I
the second-order branches of PV	Vp2	Type I
the right or left PV	Vp3	Type II
the right and left PV	Vp4	Type II
the main trunk	Vp4	Type III
the superior mesenteric vein	Vp4	Type IV

PVTT, portal vein tumor thrombus; PV, portal vein; NA, not available.

## Treatment

### Targeted Therapy

Considering the damage to liver function, limited survival benefits and patients’ drug intolerance, the traditional cytotoxic chemotherapy is not routinely recommended to HCC patients with PVTT. Targeted therapy remains the main option of systemic therapy for the patients.

Sorafenib, an oral small- molecule multi-kinase inhibitor, is the first approved targeted drug for treatment of HCC patients with PVTT based on two phase III randomized, double-blind, and placebo-controlled trials (18, 35). The MST of patients treated with Sorafenib alone was 10.7 months based on the result of the Sorafenib HCC Assessment Randomized Protocol (SHARP) study. Moreover, the MST was 6.5 months in Asia-Pacific region study, the survival time has only been prolonged for 2 – 3 months compared with placebo (18, 35, 36). In SHARP trial and Asia-Pacific population study, the stable disease (SD) and disease control rate (DCR) were 71% and 43%, 54% and 35.3%, respectively (18, 35). Bruix et al. (37) carried out an exploratory pooled analysis based on the two placebo-controlled in phase III studies. They observed that hepatitis C patients had a greater survival benefit who mainly distributed in the West. Without extrahepatic metastasis and lower neutrophil-to-lymphocyte ratio were also positive prognostic factors. The vascular invasion and high AFP were strong prognostic factors for poor outcome. In summary, sorafenib provides a survival benefit in HCC patients with PVTT but the effect is less than satisfactory.

In addition, the effect of Sorafenib in real-world clinical practice may be worse than the trials due to the selection bias. Jeong et al. (38) investigated the practical effect in 30 HCC patients with PVTT who received sorafenib monotherapy. The MST was 3.1 months and only 3 (10.0%) patients responded partially. SD and DCR were 30.0% and 33.3% respectively and were lower than the data from SHARP and Asia-Pacific trials. The common adverse events of Sorafenib are hand-foot skin reaction and gastrointestinal upset. Hepatic damage occurs occasionally, however it may lead to severe consequences (38, 39). In order to elucidate the safety and efficacy of Sorafenib monotherapy on HCC with PVTT, Kuo et al. (40) enrolled and analyzed 113 patients’ clinical data, including 56 (49.5%) Vp3 and 57 (50.5%) Vp4. The incidence rate of hepatic decompensation was 18.2% and 37% for Vp3 patients and Vp4 patients, respectively (p = 0.028). Multivariate analysis indicated that Vp4 (p = 0.041) and baseline AFP ≥ 200 ng/ml (p = 0.032) were the associated factors with hepatic decompensation. Therefore, they suggested that Sorafenib should not be recommended as the first-line treatment for Vp4 patients with higher AFP, which was consistent with the previous viewpoint by the Japan Society of Hepatology (JSH) (41). Additionally, a phase III randomized study (STORM trial) of Sorafenib as adjuvant treatment after resection or ablation for HCC indicated that Sorafenib is not an effective intervention (42). A phase III STAH trial showed that Sorafenib plus TACE tended to prolong overall survival (OS) for HCC patients with PVTT compared with Sorafenib alone, although it is not statistically significant (43).

Lenvatinib is a novel anti-angiogenesis multi-kinase inhibitor which had shown its antitumor activity against advanced HCC on the basis of a randomized phase 3 noninferiority trial (44). Compared to Sorafenib, Lenvatinib was non-inferior in MST (13.6 vs. 12.3 months, HR 0.92, 95% CI 0.79 – 1.06), which had higher objective response rate (24.1% *vs.* 9.2%, OR 3.13, 95% CI 3.59 – 7.01, p <0.0001) and longer progression-free survival (7.4 *vs.* 3.7 months, HR 0.66, 95% CI 0.57 – 0.77, p < 0.0001) with acceptable toxicity. The most common adverse events were hypertension, diarrhea, decreased appetite, and decreased weight. Recently, Lenvatinib had been approved as the first-line treatment for unresectable HCC in the European Union, America, Japan and China currently (44–46). A case of advanced HCC was reported by Takeda et al. (47), In this case, the radiological examination showed clearly portal vein invasion, after 11 months of Lenvatinib monotherapy, the PVTT was undetectable, and vascularization of the main tumor was disappeared. The patient remained alive for more than 5 years after the initiation of Lenvatinib monotherapy. This case showed that Lenvatinib monotherapy might be a considerable therapy. But there were also some toxic effects during the treatment period, such as thrombocytopenia and proteinuria. Whether the curative effect of Lenvatinib was prior to other small molecule inhibitors or not was unclear, needing further investigation and long-term observation.

Beyond Sorafenib and Lenvatinib, there are several targeted drugs that have been studied and applied clinically as the second-line therapy for HCC patients with PVTT (48). Regorafenib is the first drug which demonstrated the efficacy for Sorafenib-intolerant patients, although the MST was only 10.6 months (placebo: 7.8 months, HR = 0.63, p <0.0001) (49). Hypertension and hand–foot skin reaction were the most common grade 3 or 4 adverse events (49). Apatinib, a selective inhibitor of vascular endothelial growth factor receptor (VEGFR)-2 with low price, had shown the safety and survival benefit in HCC patients with PVTT when combined with TACE (50). At present, Hu et al. (51) attempt to perform a multicenter, open-label, randomized controlled trial to assess the efficacy and safety of stereotactic body RT (SBRT) combined with Camrelizumab and Apatinib for HCC patients with PVTT. The efficacy of Cabozantinib in the previously treated patients with advanced HCC was evaluated in a phase 3 randomized trial. The results showed that the MST of Cabozantinib group was longer than placebo group, but with higher rate of high-grade adverse events (52). Ramucirumab, an anti-VEGFR2 monoclonal antibody, has demonstrated clinical benefit for HCC patients with AFP > 400 ng/ml in the recent phase 3 trial (REACH-2) (53). The development of new drugs is advancing and finding the biomarkers to predict responses to immunotherapies is the focus of future research (54).

### TACE

TACE is considered as a standard locoregional treatment option and is widely used to treat unresectable HCC by many clinical practice guidelines (7, 19, 55). However, TACE was not administered to HCC patients with PVTT due to the potential risk of liver failure resulting from ischemia after TACE (56). The view is changing gradually with the development of medicine. Lee et al. (57) conducted a prospective controlled study and proposed that PVTT patients may benefit from TACE when the patients’ liver function was at good level (Child-Pugh A) and adequate collateral circulation around the occluded PV has been established. Then, more studies about TACE applied in PVTT patients were performed and the results are similar. Chung et al. (58) retrospectively analyzed the survival data of 125 HCC patients with PVTT from 2003 to 2007, which showed improved MST for TACE group compared to supportive care group (5.6 *vs.* 2.2 months, P < 0.001). Another two prospective studies also confirmed that TACE had more survival benefit compared with conservative treatment (7.1 *vs.* 4.1 months, P < 0.001; 8.67 *vs.* 1.4 months, P < 0.001) (59, 60). Thus, for some HCC patients with PVTT, after careful selection, those patients with good liver function and well-establishment collateral circulation might be acquire more benefits from TACE than supportive care.

Research indicates that the extent of PVTT might affect the therapeutic effect of TACE. Silva et al. (61) made a meta-analysis involving 13 trials which comprised 1,933 patients to evaluate the safety and efficacy of TACE in the treatment of HCC with PVTT. Results showed that the MST was 8 (5–15) months, the incidence of liver failure and post-treatment complications were 1% and 18%, respectively. Patients with PVTT in main portal vein trunk had worse survival than with segmental PVTT (p < 0.001), but the modified RECIST criteria response rates were similar. Xiang et al. conducted a multicenter retrospective study in 1,040 patients. The results showed that TACE could significantly improve the OS rate than the other best supportive care for type I-III patients but not type IV (62). In addition, Kim et al. (63) assessed survival data of 331 HCC patients with segmental PVTT who underwent TACE as an initial treatment, and found four risk factors were related to the dismal OS after TACE: a major tumor burden (up-to-11criteria out), extrahepatic spread, Child‐Pugh class B, and no response to TACE (stable disease or progressive disease). The study suggested that TACE should not be recommended for patients with 2 – 4 risk factors due to the poor prognosis. Yang et al. (64) retrospectively analyzed the clinical data of 379 HCC patients with PVTT who were treated with TACE as the first-line treatment, and found that patients with positive lipiodol deposition in PVTT was associated with an improved survival. In summary, for carefully evaluated HCC patients with PVTT, TACE could be a safe considerable treatment modality and the degree of lipiodol deposit in PVTT may help to assess the prognosis after TACE.

Though TACE might be an option for HCC patients with PVTT according to above researches, the efficacy of TACE alone is still limited given the MST is less than 10 months. TACE plus other treatments as a new therapeutic strategy, may improve the survival of HCC patients with PVTT. Takano et al. (65) reported a case of HCC patient with PVTT who received curative hepatectomy after TACE and sorafenib, and the disease-free survival (DFS) time was more than 12 months. A meta-analysis of 25 trials involving 2,577 patients showed that 1-year survival rate for the TACE plus RT group was significantly better than that of the TACE alone group (OR 1.36, 95% CI 1.19 – 1.54) (66). Similarly, another meta-analysis of 5 studies involving 973 patients showed that 6-month and 1-year OS rate for the TACE plus sorafenib group were significantly better than that of the TACE alone group (OR 3.47, 95% CI 2.47 – 4.89; OR 3.10, 95% CI 2.22 – 4.33). Chu et al. (67) used propensity score matching analysis to compare the effectiveness of TACE plus RT and TACE plus sorafenib groups in the treatment of HCC patients with PVTT, and found that PFS and OS did not differ significantly between these two combined strategies.

In addition, the effectiveness of TACE is associated with the embolizing agents. TACE with drug-eluting beads has been applied in clinical but its effects need more researches to support (68). Hepatic arterial infusion chemotherapy (HAIC), another locoregional treatment, much like TACE, may be another option for advanced HCC patients which showed a better response and improved prognosis compared to sorafenib in previous studies (69, 70). The conclusion was validated by a retrospective study which showed that the PFS of HCC patients with main PVTT in HAIC group was significantly longer than in sorafenib group (1.9 vs. 6.0 months, p<0.001) (71). By means of meta-analysis, Liu et al. (72) also demonstrated that HAIC is superior to sorafenib in HCC patients with PVTT, especially in type III – IV patients (Cheng’s classification). However, the study showed that HAIC was more likely to cause myelosuppression. Of note, the efficacy and safety of HAIC must be evaluated in multicenter randomized controlled trials.

### Radiation Therapy

In the past, RT was not regarded as a feasible treatment for HCC patients with PVTT because of the liver’s poor tolerance to radiation (73). But this opinion has been changed with the rapid development of precision radiotherapy technology and application of new radioisotope. Several prospective and retrospective studies have applied RT to HCC management and shown that RT could improve the prognosis, especially in patients with PVTT (74–76). The therapeutic method divided into two forms according to different administration pathways: the external beam radiation therapy and selective internal radiation therapy.

### External Radiotherapy

Advanced external radiation techniques could deliver a higher radiation dosage to the targeted regions without damage to the adjacent normal liver, including three-dimensional conformal RT (3D-CRT), intensity modulated RT (IMRT), SBRT and proton beam RT. Yu et al. (77) explored the role of external RT in the treatment of HCC patients with PVTT and showed that the objective response rate was 40% to 60% and the MST was 15 to 20 months in responders. The review presented that RT could be an effective local treatment modality. In a prospective study of Kishi et al. (78), preoperative SBRT targeting PVTT in HCC patients showed high pathological response rate and low toxicity. Postoperative RT also could improve survival outcomes for patients with resectable HCC and PVTT. Wei et al. (79) conducted an open-label randomized controlled study to evaluate the efficacy of neoadjuvant 3D-CRT in HCC patients with PVTT after hepatectomy. Results showed that the 1- and 2-years OS rates were significantly better in the neoadjuvant 3D-CRT group than the surgery-alone group (75.2% and 27.4% vs. 43.1% and 9.4%, P<0.001). Another randomized controlled trial showed that postoperative adjuvant IMRT could significantly improve the 1-, 2-, and 3-years OS rates (76.9%, 19.2%, and 11.5% *vs.* 26.9%, 11.5% and 0%, P=0.005) (80).

In clinical practice, several studies indicated that adding RT to combined treatment could improve survival for HCC patients with PVTT. Positive PVTT response to combined treatment was the most significant prognostic factor for PFS (HR 0.33, 95% CI 0.25-0.42, P < 0.001) (81). Li et al. (82) made a network meta-analysis of 15 studies involving 2,359 patients to evaluate the efficacy and safety of different modalities in patients with advanced HCC and PVTT. These modalities included SBRT, HAIC, sorafenib, TACE, SBRT plus TACE, 3D-RT plus HAIC or TACE, and TACE plus sorafenib. Results showed that RT combined with HAIC or TACE produced better survival benefit than other regimens. Im et al. (83) reported a retrospective study about 985 HCC patients with PVTT who received RT and demonstrated that RT with combined treatment is a better approach which had better OS than without combined treatment. Wu et al. (84) also suggested that compared with TACE or RT alone, RT plus TACE is a better choice in treating advanced HCC patients with PVTT. After comparing the MST of patients who received RT-TACE and TACE-RT (13.2 vs.7.4 months, P = 0.020), Li et al. (85) suggested that RT followed by TACE is a better combined therapy strategy for HCC patients with PVTT. Besides treatment methods, radiation dose is another important factor which is still controversial in clinical practice. Im et al. (83) demonstrated that the equivalent RT dose >45 Gy was a significant positive factor for OS. Due to the liver’s high sensitivity to radiation, the best radiation dose should be confirmed in further prospective studies.

### Internal Radiotherapy

Iodine-125 (^125^I) seed implantation, a type of brachytherapy, has been widely applied in treating HCC patients with PVTT and the treatment responses are favorable. Clinically, ^125^I seed implantation is always applied in the combination with TACE or portal vein stent (86, 87). Yuan et al. (87) made a meta-analysis of 8 studies involving 1,098 patients to evaluate the efficacy and safety of ^125^I seed implantation in HCC patients with PVTT. Results showed that compared with TACE alone, ^125^I seed implantation plus TACE can significantly improve patients’ survival rate (HR 0.27, 95% CI 0.14 – 0.40, p=0.000), reduce patient’s mortality risk (HR 0.46, 95% CI 0.37 – 0.54, p=0.000), and did not increase the incidence of adverse event (OR 1.07, 95% CI 0.92 – 1.25, p=0.262). The recommended dose of ^125^I is more than 110 Gy. Another retrospective study showed that combining endovascular implantation of 125I seed with stent placement, TACE, and sorafenib may provide better OS and PFS than TACE plus sorafenib in HCC patients with PVTT (88).

Transarterial radioembolization (TARE) with yttrium-90 (^90^Y) is a special treatment which successfully interweaves the microembolic procedure and RT. The available evidence showed that TARE is a safe and effective therapy for HCC patients with PVTT. The response rate ranges from 50% to 75%, and the MST is approximately 10 months (89). Two phase III trials showed that the OS of TARE and sorafenib were not significantly different (90, 91). A meta-analysis involving 17 studies showed that the 6-month and 1-year OS rate were 76% and 47% in TARE group, more than in sorafenib group (54% and 24%) (92). The incidence of grade 3 or 4 adverse events in TARE group was lower than in sorafenib group (9% *vs.* 28%, P = 0.129). Abdominal pain, nausea and fatigue were the frequent adverse events of TARE (92). Thus, the tolerance of TARE may help to recommend its clinical use. Spreafico et al. (93) found that bilirubin level, extension of PVTT and tumor burden were firmly associated with prognosis of patients with HCC and PVTT treated with TARE, and proposed to build a prognostic stratification to identify suitable candidates. The effectiveness of the prognostic model had been validated by two retrospective single-center study (94, 95), and should be further evaluated in prospective studies.

Compared with external radiotherapy, internal radiotherapy is a more invasive radiotherapy. However, internal radiotherapy has a high dose and continuous release radiation for PVTT and low damage to the nearby normal liver tissues. Especially for patients with malignant stenosis or occlusion of the portal vein, internal radiotherapy plus portal vein stent could not only greatly alleviate the portal hypertension, but also prevent the reinvasion of PVTT into the portal vein (96–98). For HCC patients with PVTT, the selection of external radiotherapy or internal radiotherapy remains unclear. In a retrospective study, Tan et al. (96) showed that internal radiotherapy plus TACE had longer OS than external radiotherapy plus TACE (13.1 vs. 8.0 months, p= 0.021). Internal radiation therapy might be more effective but also more invasive. Most of HCC patients with PVTT are at the end stage, the doctors need to evaluate the condition of specific patients carefully, to choose a better therapy.

### Surgical Resection

Liver resection is the main treatment for patients with HCC that may offer the best chance of cure (7). However, the presence of PVTT, regardless of the extent, has been viewed as a contraindication of surgery by BCLC staging system in western countries (15). Therefore, most patients lost the chance for radical operation and the possibility of cure is almost zero. However, with the advances in surgical technologies and improvements in perioperative management, aggressive surgical resection has been proposed and adopted to treat some selected HCC patients with PVTT in several center. Surgical treatment has been considered as a possible choice when the primary tumor and PVTT could be completely resected, without distant metastasis and damage to liver function (5). Hepatectomy and thrombectomy are carried out according to the location and extent of tumor and PVTT. The en bloc resection of PVTT with tumor is considered when the PVTT lies within the liver resection line (Type I – II or Vp1 – Vp3), including segmental hepatectomy and hemihepatectomy. When the PVTT lies beyond the resection line (Type III – IV or Vp4), hepatectomy plus thrombectomy could be considered. Portal vein resection and reconstruction should be performed when the PVTT invading the main portal vein wall (99–101).

Up to now, a number of studies have evaluated the efficacy of surgical treatment on the disease, especially in Asian liver centers. Kokudo et al. (102) published a large retrospective study of 6,474 HCC patients with PVTT in Japan, including 2,093 patients who underwent liver resection and 4,381 patients who received other therapeutic interventions. Results showed that the MST of surgical group was significantly longer than that of non-surgical group (2.87 vs. 1.10 years, P < 0.001) with good liver function (Child-Pugh A). A further subgroup analysis indicated that liver resection could result in survival benefits as long as the PVTT is limited to a first-order branch (Vp1 – Vp3). However, the benefit was not significant in patients whose PVTT affected the main trunk or contralateral branch (Vp4). Similar results were reported by Wang et al. (103). They retrospectively analyzed 1,580 HCC patients with PVTT from four largest tertiary hospitals in China and figured out that the treatment was an independent risk factor of OS. The MST of the surgical group for types I and II patients were 15.9 and 12.5 months respectively, significantly longer than nonsurgical counterparts. What’s more, TACE plus RT may provide more survival benefit to types III patients than surgical treatment (8.9 vs. 6.0 months, P=0.063). A similar result is obtained by Chen et al. (104). In a word, HCC patients with PVTT could benefit from surgery but the prognosis is affected by the extent of PVTT.

In order to identify which factors might affect the survival outcome, Huo et al. (99) retrospectively analyzed the clinical data of 487 HCC patients with PVTT who underwent liver reresection. Results showed that the liver function and tumor differentiation were risk factors of short-term and longer-term survival respectively, while AFP was associated with both short-term and longer-term survivals. Zhang et al. (105) developed an EHBH/PVTT scoring system to guide the HCC patients’ selections with PVTT (Vp1 – Vp3) who could benefit from negative margin (R0) liver reresection. The score was calculated by using total bilirubin (≥17.1 µmol/L=1), AFP (≥20 µg/L=2), tumor diameter (3-5 cm=1, >5 cm=2), and satellite lesions (Yes=1). Liver resection was recommended for patients when EHBH-PVTT score ≤3. After analyzing a nationwide database of 1,590 HCC patients with PVTT who underwent liver resection, Chen et al. (106) found that the actual 3-year survival rate of patients was 11.7%. The independent prognostic factors of long-term survival included total bilirubin, AFP, types of hepatectomy, extent of PVTT, intraoperative blood loss, tumor diameter, tumor encapsulation, R0 resection, liver cirrhosis, adjuvant TACE, postoperative early recurrence (< 1 year), and recurrence treatments. In addition, postoperative adjuvant TACE could improve the survival of HCC patients with PVTT (107).

The surgical technique may be an important factor which influences the prognosis. “Liver resection first” is the most common major operation performed on HCC patients, PVTT is often removed after hepatectomy in previous studies which concluding that type III/IV PVTT patients were unable to gain a survival advantage through surgery. Ban et al. (108) performed tumor thrombectomy prior to the hepatectomy for 19 Vp4 patients. The 3- and 5-year OS rates in the study were 41.8% and 20.9% respectively, which were significantly higher than in other studies. Peng et al. (100) put forward a concept of “thrombectomy first”, which means the PVTT should be removed prior to liver resection when it is located in the main PV, the bifurcation or the contralateral PV. They subsequently shared three types III/IV (Vp4) cases which were treated with “thrombectomy first” method and achieved good long-term survival, the DFS were 13, 9 and 4.6 years respectively (100). The new surgical technique may improve the management of HCC patients with PVTT, especially for type III/IV PVTT patients. The efficacy of “thrombectomy first” approach should be further validated in multi-center and randomized trials.

### Liver Transplantation

Compared to liver resection, LT can not only completely resect the lesion but also restore liver function. As a curative treatment for HCC patients, the indication of LT is expanding. Lots of studies indicated patients beyond the conventional Milan criteria are also suitable for LT, but in most studies, PVTT remains as an absolute contraindication due to the high rate of recurrence and poor prognosis (109–111). In recent years, several centers tried to do LT in HCC patients accompanied by PVTT, and the clinical data have shown that LT can provide survival benefit for selected HCC patients with PVTT. Herein, we reviewed the related literature and crested a summary in **Table 2**.

**Table 2:** Liver transplantation for HCC patients with PVTT.

Author, Year	Country	Study design	N (Enrollment Period)	Treatment	Downstaging before LT	Classification of PVTT (n)	Survival time	DFS rate(1-,3-,5-year)	OS rate(1-,3-,5-year)
Yang, 2020 (112)	China	Retrospective study	75 (2016-2018)	DDLT	NA	Vp2-3 (47)	NA	44.4%,40.0%, NA	74.1%, 65.4%, NA
						Vp4 (28)	NA	28.6%,21.4%, NA	64.3%, 30.6%, NA
Assalino, 2020 (113)	Switzerland	Retrospective study	30 (2004-2018)	DDLT/LDLT	Yes	Vp1 (7); Vp2 (12); Vp3 (5); Hepatic vein (6)	NA	63.3%, 56.3%, 56.3%	76.7%, 66.2%, 59.6%
Soin, 2020 (114)	India	Prospective study	46 (2006-2017)	LDLT	Yes	Vp1 (1); Vp2 (12); Vp3 (11); Vp4 (1)	NA	77%, 77%, 51%	82%, 57%, 57%
					No	Vp1 (5); Vp2 (13); Vp3 (3); Vp4 (0)	NA	63%, 48%, 40%	80%, 59%, 48%
Jeng, 2018 (115)	China	Case report	1 (2013)	DDLT	Yes	Type II	DFS is more than 20 months	NA	NA
Levi, 2017 (116)	Italy	Case series	4 (2002-2015)	DDLT	Yes	Vp1 (3); Vp3 (1)	Median DFS: 39.1 (6–76) months	NA	NA
Lee, 2017 (117)	Korea	Retrospective study	11 (2009-2013)	LDLT	Yes	Vp3 (3); Vp4 (1)	Mean DFS: 8.3 (1-20) months	63.6%, 45.5%, 45.5%	72.7%, 63.6%, 63.6%
					No	Vp2 (3); Vp3 (1); Vp4 (3)			
Jeong, 2017 (118)	Korea	Retrospective study	17 (2007-2014)	LDLT	Yes	Vp2 (7); Vp3 (7); Vp4 (1); Hepatic vein (2)	NA	70.6%, 57.8%, NA	87.45%, 60.5%, NA
Choi, 2017 (119)	Korea	Retrospective study	34 (2005-2015)	LDLT	NA	Type I (27)	NA	68.2%, 63.9%, 63.9%	85%, 60.3%, 50.3%
						Type II (7)	NA	28.6%, 14.3%, 14.3%	71.4%, 14.3%, 14.3%
Han, 2016 (120)	Korea	Retrospective study	8 (2011-2012)	LDLT	Yes	Type II, Type III	MST: 33 (22–48) months	87.5%, NA, NA	NA
Ettorre, 2010 (121)	Italy	Case report	1 (2009)	DDLT	Yes	Type II	survival for more than 4 years	NA	NA
Zhou, 2011 (122)	China	Retrospective study	12 (2003-2010)	DDLT	No	Type II (6); Type III (6)	MST: 7 months	NA	30.0%, 10.0%, NA
Wang, 2010 (123)	China	Retrospective study	62 (2001-2007)	DDLT	NA	Type I_0_ (12); Type I-III (50)	NA	29.6%, 13.4%, NA	NA
Xu, 2004 (124)	China	Retrospective study	24 (1999-2003)	DDLT	NA	Type II (14); Type III (10)	MST: 8 months	29.5%, NA, NA	23.2%, NA, NA

PVTT, portal vein tumor thrombus; DDLT, deceased donor liver transplantation; LDLT, living donor liver transplantation; MST, median survival time; DFS, disease free survival; OS, overall survival; NA, not available.

Xu et al. (124) considered that LT was an efficient treatment but palliative treatment for HCC patients with PVTT. They retrospectively analyzed the survival data of 24 HCC patients with PVTT who received deceased donor LT (DDLT), and compared it with 27 patients who underwent liver resection. The OS rates at 6-month, 1-and 2-year were 66.7%, 29.5% and 23.6% for the LT group, and 33.3%, 22.2% and 14.8% for the resection group (P=0.0335), respectively. But the tumor recurrence rate was as high as 66.7% for the LT group. Zhou et al. (122) compared the therapeutic effects of LT and other therapies on HCC patients with PVTT. Results showed that the 1-, 3-year OS rate in LT group were 30% and 10%, which was better than the conservative treatment (12% and 4%), but inferior to resection combined with adjuvant chemotherapy (70% and 20%). Our previous study showed that pre-transplant AFP level and 18 F-FDG standard uptake value (SUV max) were independent risk factors for HCC recurrence fonc.2021.635731. The study also proposed that patients with AFP < 1000 ng/mL and SUV max < 5 might be suitable for LT.

Given the shortage of donor organs, DDLT is still limited in the treatment of HCC patients with PVTT. In recent years, the number of living donor LT (LDLT) is increasing, which provided a therapeutic option for curing HCC patients with PVTT. Choi et al. (119) retrospectively analyzed 34 HCC patients with PVTT who underwent LDLT. The 1-, 3- and 5-year OS and DFS rates for segmental PVTT group were 85%, 60.3%, 50.3% and 68.2%, 63.9%, 63.9%, respectively, which were higher than lobar PVTT group (71.4%, 14.3%, 14.3% and 28.6%, 14.3%, 14.3%, respectively). They proposed that segmental PVTT could benefit from LT, especially when the AFP level less than 100 ng/mL. Similar result was reported by Lee et al. (117). The 5-year OS rates and DFS rates were 63.6% and 45.5% in their study. They proposed that PVTT is not an absolute contraindication for LDLT. LDLT was considered to be a curative treatment option when the PVTT did not extend into the main PV and the multiplication of AFP and protein induced by vitamin K absence/antagonist-II (PIVKA) score is less than 20000. Therefore, LT can improve the survival of HCC patients with PVTT, especially for carefully selected recipients.

Bridging treatment before LT could help HCC patients with PVTT downstage to meet the qualifications for LT, such as TACE, HAIC, TARE, CCRT (125). Chapman et al. (126) reported 17 HCC patients with macrovascular invasion underwent LT after successful downstaging to within the Milan criteria through TACE. The result was satisfied, the 5-year OS rate was up to 93.8%. Levi Sandri et al. (116) reported 4 patients in BCLC stage C received TARE with ^90^Y before LT. Result showed patients had a complete response for the PVTT and eventually accepted LT, the median DFS was 39.1 months. A similar case reported by Ettorre et al. (121, 125) showed that an HCC patient with PVTT was successfully downstaged through TARE and received LT, then survived for more than four years. Another typical case reported by Jeng et al. (115) showed that an HCC patient with tumor thrombus invading right main PV received DDLT after successful downstaging by multimodal treatments, and the survival time was more than 20 months without tumor recurrence or metastasis. Assalino et al. (113) conducted a multi-center retrospective cohort study and demonstrated that HCC patients could be considered for LT when the vascular invasion achieved radiological complete regression after locoregional therapies and the pretransplant AFP < 10 ng/ml.

Downstaging treatment is also suitable for LDLT. Han et al. (120) reported 8 HCC patients with PVTT who accepted LDLT after successful downstaging of tumor through CCRT and HAIC. The MST was 33 months. Moreover, Jeong et al. (118) reported 17 HCC patients with major vascular invasion who received LDLT after combined treatment modalities. The DFS rates and OS rate at 1- and 3-year were 70.6% and 57.8%, 87.4 and 60.5%, respectively. Recently, Soin et al. (114) shared treatment experience with LDLT in HCC patients with PVTT. Compared to the patients without the downstaging before LDLT, the 1-, 3- and 5-year DFS rates were improved in patients with successful downstaging (77%, 77%, and 51% *vs.* 63%, 48%, and 40%, P=0.35), although without statistical significance. Taken together, these results demonstrate that the downstaging could actually improve survival of HCC patients with PVTT before LT.

All in all, LT could be a promising treatment modality for HCC patients with PVTT. Downstage treatment for these patients is quite important. Combined therapy before LT seems to play an important role in the downstaging strategy for LT candidates. However, the number of related studies is still less. More prospective studies and randomized controlled trials are needed to assess the application value of LT in HCC patients with PVTT. In addition, it is urgently necessary to develop a scoring system to identify suitable candidates for LT.

### Other Strategies

Besides, with the development of immunotherapy in the area of cancer therapy, the combination of small molecular targeted therapy and immunotherapy might be a promising direction. Programmed death 1 (PD­1) inhibitors have gained great success in some types of cancer treatment. For hepatocellular carcinoma treatment, PD-1 inhibitors showed promising clinical activity in phase 1/2 studies (127, 128). However, the response rates were range of 15-20% in single-agent treatment studies, they did not improve overall survival, either (129, 130). It has been reported that anti­VEGF therapies could reduce VEGF­mediated immunosuppression within the tumor and its microenvironment (131–133). So, anti-VEGF therapies might also enhance the anti PD-1 or anti PD-L1 efficacy by reversing immunosuppression in tumor (134, 135). Bevacizumab, a monoclonal antibody, which targets VEGF (136), inhibits angiogenesis, and showed response rates of 13 to 14% in single agent phase 2 studies (137–140). Atezolizumab, which targets PD­L1 to prevent interaction with receptors PD­1 and B7­1, activate T-cell in immunotherapy. The combination of atezolizumab and bevacizumab showed a promising antitumor ability with acceptable side effect in treatment of untreated unresectable hepatocellular carcinoma. The reported response rate was 36%, and the median progression free survival was 7 months (141). Another global, multicenter, phase 3 randomized trial, IMbrave150 showed us inspiring results. Compare to sorafenib treatment alone, the overall survival at 12 months was 67.2% in combo therapy group, but 54.6% in sorafenib group, median progression­free survival was 6.8 months (95% CI, 5.7 – 8.3) and 4.3 months (95% CI, 4.0 – 5.6), respectively. Grade 3 or 4 adverse events occurred in 56.5% of 329 patients who received at least one dose of atezolizumab-bevacizumab and in 55.1% of 156 patients who received at least one dose of sorafenib. Serious adverse events occurred more frequently with atezolizumab-bevacizumab (125 patients,38.0%) than with sorafenib (48 patients, 30.8%) (142). Though atezolizumab plus bevacizumab therapy prolong overall survival and PFS in unresectable hepatocellular carcinoma patients, the high rate of serious side effects needs to be on the alert.

## Conclusion

In conclusion, PVTT remains as the blockage in the treatment of HCC, which contributes in the high recurrence rate and poor prognosis. Besides Sorafenib and Lenvatinib, no other standard treatment regimen is currently available for HCC with PVTT. For these patients with HCC and PVTT, the surgery, TACE, RT and various combination therapies were effective and safety choices, which could help to prolong the survival time and promote the quality of life. LT may be a curative treatment option for highly selected patients, especially LDLT. In the future, larger scale randomized trials are needed to develop better treatment strategy to manage HCC patients with PVTT.

## Author Contributions

FL and ML collected related papers and drafted the manuscript. FL drafted the figures. JD participated in the design of the review. SZ was responsible for the supervision of the work. All authors contributed to the article and approved the submitted version.

## Funding

Innovative Research Groups of National Natural Science Foundation of China (No. 81721091), National S&T Major Project (No. 2017ZX10203205), Zhejiang International Science and Technology Cooperation Project (NO.2016C04003), Research Unit Project of Chinese Academy of Medical Sciences (2019-I2M-5-030), and Grant from Health Commission of Zhejiang Province (JBZX-202004).

## Conflict of Interest

The authors declare that the research was conducted in the absence of any commercial or financial relationships that could be construed as a potential conflict of interest.

## Publisher’s Note

All claims expressed in this article are solely those of the authors and do not necessarily represent those of their affiliated organizations, or those of the publisher, the editors and the reviewers. Any product that may be evaluated in this article, or claim that may be made by its manufacturer, is not guaranteed or endorsed by the publisher.
